# Thoracic Organ Doses and Cancer Risk from Low Pitch Helical 4-Dimensional Computed Tomography Scans

**DOI:** 10.1155/2018/8927290

**Published:** 2018-09-24

**Authors:** Chengwen Yang, Ransheng Liu, Xin Ming, Ningbo Liu, Yong Guan, Yuanming Feng

**Affiliations:** ^1^Department of Biomedical Engineering, Tianjin University, Tianjin, China; ^2^Department of Radiation Oncology, Tianjin Medical University Cancer Institute & Hospital, Tianjin, China; ^3^School of Biomedical Engineering, Tianjin Medical University, Tianjin, China

## Abstract

**Purpose:**

To investigate the dose depositions to organs at risk (OARs) and associated cancer risk in cancer patients scanned with 4-dimensional computed tomography (4DCT) as compared with conventional 3DCT.

**Methods and Materials:**

The radiotherapy treatment planning CT image and structure sets of 102 patients were converted to CT phantoms. The effective diameters of those patients were computed. Thoracic scan protocols in 4DCT and 3DCT were simulated and verified with a validated Monte Carlo code. The doses to OARs (heart, lungs, esophagus, trachea, spinal cord, and skin) were calculated and their correlations with patient effective diameter were investigated. The associated cancer risk was calculated using the published models in BEIR VII reports.

**Results:**

The average of mean dose to thoracic organs was in the range of 7.82-11.84 cGy per 4DCT scan and 0.64-0.85 cGy per 3DCT scan. The average dose delivered per 4DCT scan was 12.8-fold higher than that of 3DCT scan. The organ dose was linearly decreased as the function of patients' effective diameter. The ranges of intercept and slope of the linear function were 17.17-30.95 and -0.0278--0.0576 among patients' 4DCT scans, and 1.63-2.43 and -0.003--0.0045 among patients' 3DCT scans. Relative risk of cancer increased (with a ratio of 15.68:1) resulting from 4DCT scans as compared to 3DCT scans.

**Conclusions:**

As compared to 3DCT, 4DCT scans deliver more organ doses, especially for pediatric patients. Substantial increase in lung cancer risk is associated with higher radiation dose from 4DCT and smaller patients' size as well as younger age.

## 1. Introduction

Four-dimensional computed tomography (4DCT) which is an advanced technique to acquire a sequence of 3DCT with respect to respiration signal which could be used to monitor the lesion motion in patients has been widely utilized in the radiation therapy as well as diagnostic arena [1]. 4DCT data could be applied to contour moving target, such as the clinical target volume to determine the internal target volume and observe the intrafractional motion of organs and lesions in the thoracic and abdominal regions across the treatment. To overlap the motion of tissues due to respiration, 4DCT allows a highly oversampled CT data acquisition, resulting in a rapid increase in radiation dose to the organs at risk (OARs). Effective organ doses delivered from a 4DCT scan have been measured and estimated by a few research groups [2–5]. Yet, to our knowledge, no data of patient-specific imaging dose from 4DCT protocols have been directly reported. Monte Carlo simulation has been regarded as the golden standard to compute the patient-specific imaging dose in CT and cone beam CT scans [6–10]. In our study, we quantified and compared radiation dose to OARs in 4DCT scans with conventional 3DCT scans using Monte Carlo simulation and investigated the imaging dose as function of patient size. The estimated relative risk of cancer incidence was also calculated with the National Research Council Biologic Effects of Ionizing Radiation (BEIR) VII report. 

## 2. Methods and Materials

### 2.1. Patients Characteristics

With the Institutional Ethnics Committee approval (CRTOG1601) and patient consent, radiotherapy treatment planning (RTP) CT images, organ contours, age, and gender of 102 cancer patients (51 males and 51 females) treated from the year of 2007 to 2017 in our institution were used in this retrospective study. The average age of patients at diagnosis was 65 (range, 6-93). The volumes of OARs were segmented using the Pinnacle^3^ RTP system (Philips Healthcare, Best, Netherlands). Patient effective diameter was computed at the nipple level from body contours using DICOMan software [11], which ranged from 184.50 mm to 465.10 mm.

### 2.2. Data Acquisition

A commercially available 16-slice Brilliance Big Bore CT scanner (Philips Medical System) in our clinic was used. Patients were set up in supine position and immobilized with Body Pro-Lok immobilization device (CIVCO Medical Solutions, Coralville, IA, USA) during data acquisition. For each patient, thoracic 4D helical and 3D axial scans were acquired with the collimation of 16 × 0.75 mm, 16 × 1.5mm, and 8 × 3 mm. Varian real-time position management system v1.7.5 (RPM, Varian Medical Systems, Palo Alto, CA, USA) was used in the 4DCT acquisition. The scan protocol was set as 120 kV and 100 mAs. The pitch of helical mode is 0.059 and rotation time is 0.44s.

The volumes of OARs within the primary beam were segmented using the Pinnacle^3^ RTP by one experienced radiation oncologist and confirmed by another experienced radiation oncologist. The following OARs were defined: heart, bilateral lungs, spinal cord, trachea, and esophagus. Averaged intensity projection (AveIP) was used in OAR definition in the 4DCT. With the aid of DICOMan, the images and structures of the patients were converted into EGS4 CT phantoms based on scanner-specific Hounsfield units to density conversion.

### 2.3. Monte Carlo Simulation

The Monte Carlo method was used to simulate the kV X-ray beam of 3DCT and 4DCT with the thoracic protocols. The source model of Philips Brilliance Big Bore 16-slice CT scanner was calibrated and validated through measurement in our previous work [12]. The proposed source model consists of an extended circular source located at the X-ray target level, with its characteristics defined by the energy spectrum, source distribution, and fluence distribution. An in-house C++ code was developed to perform an automatic beam commissioning to generate the source model based on a set of measurement data, including central axis PDD distribution in water, the dose profiles along lateral and longitudinal directions at isocenter level, and in-air beam output measured at isocenter through a series of cylindrical cones. The detailed derivations between the measurement data and source models were described in the previous work [12]. An EGS4/BEAM Monte Carlo code, MCSIM, was employed to reconstruct the photon beams from the generated source model and to calculate the dose distributions in water and two CTDI phantoms [13–15]. In Monte Carlo simulations, the energy cutoff for electrons (ECUT) and photons (PCUT) and the energy threshold for *δ*-ray production (AE) and bremsstrahlung production (AP) were set as ECUT = AE = 521 keV and PCUT = AP = 10 keV, respectively. The number of histories was 500,000 and the calculation time was 2-4 h for each Monte Carlo simulation in order to achieve a statistical uncertainty (1*σ*) of less than 2%. The benchmark results of EGS4/MCSIM have been reported previously [16].

In the axial scan mode of 3DCT, a series of 12 coplanar fields around the gantry rotation axis with an interval of 30° were simulated to mimic the axial mode of CT acquisition. After each 360° gantry rotation, the phantom isocenter moved by a certain distance equivalent to the table movement. In the helical scan mode of 4DCT, the pitch value was considered in computing the incremental table movement (i.e., isocenter movement) between two consecutive gantry fields.

To convert Monte Carlo simulation into absolute dose, absorbed doses were first measured at the isocenter of a CTDI phantom (16 cm in diameter) following the AAPM TG-61 protocol with a calibrated EXRADIN A12 ionization chamber (Standard Imaging, Middleton, WI, USA) for the thoracic and abdominal scan protocols in 3DCT and 4DCT. The CTDI phantom was also scanned and the images were imported into treatment planning system and converted into digital phantom for Monte Carlo simulation with MCSIM. Monte Carlo simulation was then performed to a chamber volume inside the phantom with the same beam setups using the proposed source model. The ratios of Monte Carlo simulation values and measured absorbed doses of the same phantom yielded conversion factors, which were used in the absolute dose calculations in the patient's anatomy.

### 2.4. Organ Dose and Risk Estimation

We calculated organ doses for 102 patients from the 3DCT and cine 4DCT by using Monte Carlo simulation. Doses delivered to the OARs were calculated as the mean doses of all voxels within the defined structures. Then the doses from 3DCT and 4DCT were fitted against patient effective diameter to investigate the relationship between the patient size and imaging dose from CT scanner.

One set of patient data was used for estimating dose difference calculated with different phantoms generated from maximum intensity projection (MIP) and AveIP. The OARs were contoured and confirmed by the same radiation oncologists following the same criterion on both MIP and AveIP.

In this study, the function of estimated relative risk (ERR) in BEIR VII models was used in calculation of the cancer risk in female lung cancer and male lung cancer [17]. The ERR was defined in BEIR VII report as follows:

(1) $$\begin{array}{c} ERR\left(\;e,a\;\right)=\beta_{s}\times D\times \exp\left(\;\gamma e^{*}\;\right)\times {\left(\;\frac{a}{60}\;\right)}^{\eta } \end{array}$$

where *e* is patient's age at exposure in years, a is attained age (years), D is the radiation dose (Sv), *e*^*∗*^is(*e* − 30)/10for*e* < 30and zero for e ≥ 30, and *β*_*s*_ is the gender- and site-specific parameter (95% confidence interval) as shown in Table 12-2 of BEIR VII report as 0.32 (0.15, 0.70) for male and 1.40 (0.94, 2.1) for female. *γ* and* η *equal -0.30 and -1.4 for lung cancer, respectively.

## 3. Results

### 3.1. Organ Dose Distribution for 3DCT and 4DCT

**Figure 1:**
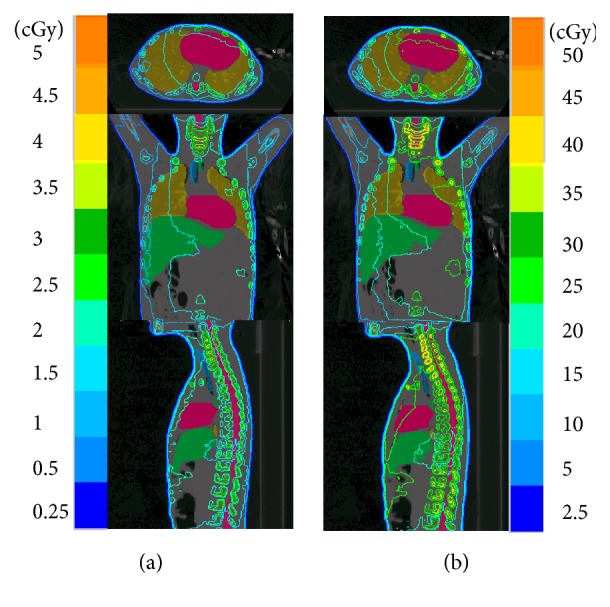
Dose distribution of a pediatric patient delivered by one scan of (a) 3DCT (120kV, 100mAs) and (b) 4DCT (120kV, 100mAs).

To depict the dose distribution in patients from 3DCT and 4DCT scans, a pediatric patient was exemplified with different isodose lines in Figure 1, which shows that dose gradient distributions in the 3DCT and 4DCT were inhomogeneous. The hot spot concentrated in the region of the head and neck.

In the 102 patients, the average of mean dose to heart, bilateral lungs, spinal cord, esophagus, trachea, and skin was 0.8 (±0.25), 0.71(±0.24), 0.74 (±0.21), 0.79 (±0.24), 0.85 (±0.34), and 0.64 (±0.17) cGy in one3DCT scan, while that in one 4DCT scan was 10.3 (±3.03), 9.46 (±2.44), 9.72 (±2.54), 10.37 (±3.13), 11.84 (±3.22), and 7.82 (±1.58) cGy. The mean dose delivered to whole body per 4DCT scan was 12.8-fold higher as compared with that of per 3DCT scan.

The differences of estimated dose between AveIP- and MIP-based simulations are shown in Table 1. The ratios of these two type simulations were 1.12-1.15.

### 3.2. Correlation of Organ Doses and Patient Size

**Figure 2:**
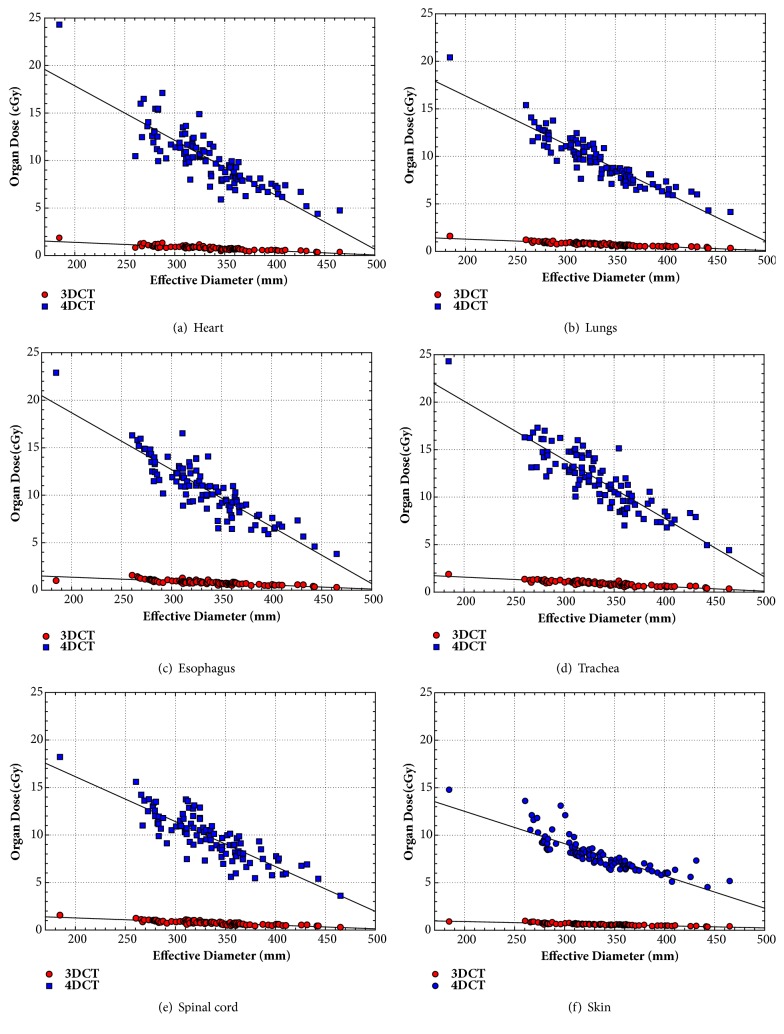
The mean doses to (a) heart, (b) lungs, (c) esophagus, (d) trachea, (e) spinal cord, and (f) skin decreased monotonically with increasing patients' effective diameter for 3DCT and 4DCT scans.

For 3DCT and 4DCT scans, the mean doses deposited to the various organs decreased with the increasing patients' effective diameters precalculated as shown in Figure 2. The dose from 4DCT scan was much higher than from 3DCT scan for patients' heart (a), lungs (b), esophagus (c), trachea (d), spinal cord (e), and skin (f). The linear correlation between organ dose and patient size was derived and the following function of organ dose was obtained.

(2) $$\begin{array}{c} Organ\;\;Dose=D+a\times Effective\;\;Diameter \end{array}$$

The parameters (D, a) and the coefficient of determination R^2^ were (2.43, -0.0045, 0.70) and (30.95, -0.0574, 0.67) for trachea, (2.04, -0.0039, 0.84) and (25.90, -0.0491, 0.83) for lungs, (1.91, -0.0035, 0.61) and (24.24, -0.0435, 0.60) for spinal cord, (2.21, -0.0042, 0.67) and (29.17, -0.0559, 0.67) for esophagus, (2.27, -0.0044, 0.73) and (29.43, -0.0576, 0.71) for heart, and (1.63, -0.003, 0.69) and (17.17, -0.0278, 0.79) for skin per 3DCT and 4DCT scan, respectively.

### 3.3. Estimated Risk from CT Scans

**Figure 3:**
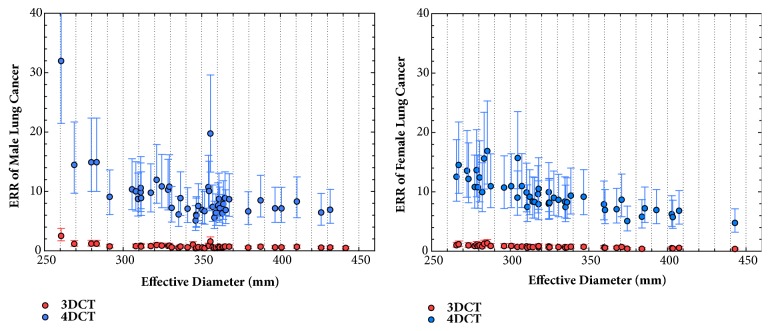
Estimated relative risks for (a) male and (b) female lung cancer from 3DCT and 4DCT scans. The upper and lower bars indicate the 95% confidence intervals (CI).

The estimated relative risk that incorporates the magnitude of radiation exposure, sex, and patient age at the time of exposure in BEIR VII report was computed. Sex-specific estimated relative risk of lung cancer was shown in Figure 3. The relative risk for 4DCT scan decreased generally with increasing patients' effective diameter, similarly as for 3DCT scan. Yet the relative risk for 4DCT scan was much higher than that for 3DCT scan with a ratio of 15.68:1.

## 4. Discussion

4DCT has been used widely in radiation oncology for RT planning which helps reduce the chances of having a geographic miss and increases the chances of local control. 4DCT-scan protocols are designed to produce a highly oversampled CT data set with assistance of a low pitch scan to cover the patients' respiratory motion, which would involve a steep increase in radiation dose. This work quantifies the dose distribution and demonstrates the relationship between the patient size and organ dose.

With the parameter settings, the thoracic organ doses for 4DCT were much higher than that for 3DCT (7.82-11.84 cGy versus 0.64-0.85 cGy after normalization to 100 mAs). In helical mode, 4DCT-scan protocols attempt to account for the patient's respiratory motion by utilizing a low pitch scan (e.g., pitch = 0.1) to produce a highly oversampled CT data set [2]. Dose increase resulting from the highly oversampled scan is inversely proportional to the table pitch. On a per mAs basis, utilization of a pitch of 0.1 could lead to an approximately 10-fold dose increase relative to a pitch of 1.0. Similarly for oversampled data acquisition, effective organ dose measured with cine mode in 4DCT scan was four times higher than those with conventional 3DCT [3]. In the risk model for lung cancer, 4DCT scan could involve more relative risk than conventional CT (with a ratio of 15.68:1). Considering the conclusion by Darby et al. [18], the patients who deposited more heart dose have relatively higher risk of ischemic heart disease.

Other studies on 4DCT reported that the radiation dose depends intensively on the setting of different protocols. Effective doses measured in adult anthropomorphic phantoms were 6.14 cGy for lung (3) in cine mode, 5.72 cGy for lung, and 5.05 cGy for esophagus (4) in helical mode with the pitch of 0.125, 5.34 cGy for lung, and 8.73 for esophagus (5) in helical mode with the pitch of 0.516. The scan setups were 120 kV and 100-120 mA. All these effective doses were measured in anthropomorphic phantoms (54-74 kg) by metal oxide semiconductor field effect transistor (MOSFET) dosimeters or thermoluminescence dosimeters (TLD). Although these effective doses did not reflect the accurate dose distributions in the real patients' body, it was concluded that lower pitch could involve the more dose delivered to the OARs in helical 4DCT. For dose estimation, DeMarco* et al*. [2] conducted the Monte Carlo simulation on a GSF voxel phantom and concluded that average lung dose was a function of tube potential. Their reported lung dose is 15 cGy at 100 mAs and double pitch, slightly higher than our results (4.15-14.10 cGy) in this study.

The converted phantoms from patients CTs in this study could not be used to mimic the dynamic respiratory motion. The differences of estimated dose resulting from AveIP and MIP were in consistent with the one from a research of MV-beam treatment [19] and the MIP-based simulation would overestimate the dose delivered. The AveIP-based simulation was recommended in 4DCT Monte Carlo simulation for the comprehensive consideration of respiratory motion.

The helical 4DCT with low pitch could result in 10% speed-up in scanning but 92% dose efficiency and the broadening of slice sensitivity profile from 1.25 to 2.3 mm on the 16-slice system [20]. Compared with cine 4DCT, helical 4DCT leads to doses of approximately 1.5-fold higher (43.5 mSv/28.8 mSv) [4]. Cautions should be taken when a helical 4DCT is to be used and imaging dose is a concern.

It should be noted that dose from 4DCT or 3DCT scans is much less than the treatment dose delivered in radiation therapy to lung tumor (generally in the range of 45-70 Gy) and the normal tissue dose constrains in conventionally fractionated radiotherapy (mean dose of less than 20-26 Gy) [16]. Nevertheless, accumulated dose to normal tissues from 4DCT scans should still be considered to minimize the potential relative risk.

## 5. Conclusion

This study with data of 102 patients demonstrated a strong inverse correlation between patient effective diameter and mean organ dose to the thoracic organs. The average dose delivered per 4DCT scan was 12.8-fold higher than that per 3DCT scan. Substantial increase in lung cancer risk is associated with higher radiation dose from 4DCT and smaller patients' size.

## Figures and Tables

**Table 1:** Differences of estimated dose between AveIP- and MIP-based simulations.

	AveIP		MIP		
	Volume (cc)	Dose (cGy)	Volume (cc)	Dose (cGy)	Ratio
Heart	524.28	7.02	558.03	7.87	1.12
Lungs	1682.19	7.38	1680.46	8.25	1.12
Spinal Cord	9.16	6.67	8.81	7.65	1.15

AveIP = averaged intensity projection; MIP = maximum intensity projection.

## Data Availability

The data used to support the findings of this study are included within the article.
